# Animal Vaccination

**Published:** 1867-08

**Authors:** 


### *Animal Vaccination.*

The French Academy of Medicine, the body to which is assigned the management of the public and gratuitous vaccinations in Paris, and the distribution of lymph, has been for some time past engaged in practically investigating the claims of the practice of "animal vaccination," introduced into France from Nadles by M. Lanoix. At the last meeting of this learned body, M. Depaul read his report on the results of the investigation, the chief conclusions of which we subjoin. 1. The transmission of cow-pock by its inoculation from heifer to heifer is obtained without difficulty. 2. No accident occurred to any of the heifers from the fact of inoculation. 3. The three first heifers were inoculated by the Naples cow-pock, and forty-two others by that obtained from Beaugency. 4. The cow-pock has lost none of its properties through successive inoculations. 5. The course of the eruption has been more rapid on the heifer than on the human subject, the pimple appearing on the third day, and suppurating in the course of the seventh or eighth. 6. In sick heifers the pustules presented less development than in healthy ones. 7. The eruption was entirely confined to the inoculated spots. 8. Little or no reaction was observed. 9. It would be easy, especially in great centres of population, to organize services for animal vaccination. 10. The quantity of lymph which each heifer can supply is sufficient for the most extensive service. 11. According to our experiments, syphilis is not conveyable to the bovine species by inoculation. 12. When taken under proper conditions, this lymph succeeds as frequently as lymph derived from an infant. 13. Taken later than the seventh day, it produces less satisfactory results. 14. It is not unusual after inoculation of infants with the cow-pock to find the portion of incubation prolonged, the eruption not manifesting itself until between the ninth and twelfth days. 15. Sometimes on the same individual the pustules pursue an unequal and irregular course. 16. The pustules obtained by this cow-pock are more voluminous, and the phenomena of general reaction, especially at the period of suppuration, are more sensible than after human vaccination. 17. These manifestations have never taken on a serious character in any of the infants inoculated by us. 18. The number of resulting pustules is alike in both kinds of vaccination. 19. A single puncture has sometimes given rise to the appearance of one, two, three, or even four pustules—a phenomenon which is of much rarer occurrence after human vaccination. 20. The cow-pock, like the lymph from the infant, often fails when it has been preserved between glasses or in tubes, and in this respect human vaccination seems to possess some advantage over animal vaccination. Still, the cow-pock lymph which has been kept in tubes during a month has been successfully used, and specimens which have been sent to the provinces and abroad have furnished satisfactory results. 21. We have made too few trials of cow-pock for revaccination to be able to form an opinion. 22. During the prevalence of epidemics of smallpox, one or more inoculated heifers might be sent into the districts, which would supply all the lymph necessary for the vaccinations and revaccinations.—*Med. Times and Gazette.*

April 20, 1867.

